# Fast and Accurate Motion Correction for Two-Photon Ca^2+^ Imaging in Behaving Mice

**DOI:** 10.3389/fninf.2022.851188

**Published:** 2022-04-26

**Authors:** Weiyi Liu, Junxia Pan, Yuanxu Xu, Meng Wang, Hongbo Jia, Kuan Zhang, Xiaowei Chen, Xingyi Li, Xiang Liao

**Affiliations:** ^1^Brain Research Center and State Key Laboratory of Trauma, Burns, and Combined Injury, Third Military Medical University, Chongqing, China; ^2^Center for Neurointelligence, School of Medicine, Chongqing University, Chongqing, China; ^3^Advanced Institute for Brain and Intelligence, Guangxi University, Nanning, China; ^4^Brain Research Instrument Innovation Center, Suzhou Institute of Biomedical Engineering and Technology, Chinese Academy of Sciences, Suzhou, China

**Keywords:** two-photon Ca^2+^ imaging, motion correction, behaving mice, image density feature, image registration, online experiment

## Abstract

Two-photon Ca^2+^ imaging is a widely used technique for investigating brain functions across multiple spatial scales. However, the recording of neuronal activities is affected by movement of the brain during tasks in which the animal is behaving normally. Although post-hoc image registration is the commonly used approach, the recent developments of online neuroscience experiments require real-time image processing with efficient motion correction performance, posing new challenges in neuroinformatics. We propose a fast and accurate image density feature-based motion correction method to address the problem of imaging animal during behaviors. This method is implemented by first robustly estimating and clustering the density features from two-photon images. Then, it takes advantage of the temporal correlation in imaging data to update features of consecutive imaging frames with efficient calculations. Thus, motion artifacts can be quickly and accurately corrected by matching the features and obtaining the transformation parameters for the raw images. Based on this efficient motion correction strategy, our algorithm yields promising computational efficiency on imaging datasets with scales ranging from dendritic spines to neuronal populations. Furthermore, we show that the proposed motion correction method outperforms other methods by evaluating not only computational speed but also the quality of the correction performance. Specifically, we provide a powerful tool to perform motion correction for two-photon Ca^2+^ imaging data, which may facilitate online imaging experiments in the future.

## Introduction

Two-photon microscopy is widely used for investigating neural functions at diverse scales, from the size of a single spine to a neuronal population (Dombeck et al., 2007; Grienberger and Konnerth, 2012; Huber et al., 2012). Recent advances in optical imaging techniques have enabled the monitoring of neural activity with high temporal resolution. However, movement of the brain, which is generally induced by behaviors such as licking and limb movements, remains a serious limitation when recording animals that are awake (Greenberg and Kerr, 2009). Therefore, correction for brain movement enables the accurate analysis of morphology and activity for individual neurons in imaging data (Chen et al., 2013). In addition, when performing real-time processing during online functional imaging and photostimulation experiments (Griffiths et al., 2020), data processing must be performed with a high speed and robust performance. Therefore, a fast and accurate processing method for removing the movement artifacts from imaging data is a key requisite for online experiments (Mitani and Komiyama, 2018; Pnevmatikakis, 2019).

To reduce the image distortions caused by brain movements, frame-by-frame motion correction approaches have been developed based on image registration. An important set of image registration methods is based on image intensity. These algorithms are principally intuitive and provide good performance, but they are computationally expensive. As a representative intensity-based method, TurboReg (Thevenaz et al., 1998) has been integrated in ImageJ software and is widely used for correcting lateral motion artifacts. Moreover, to implement non-rigid transformation for the motion correction of Ca^2+^ imaging data, NoRMCorre (Pnevmatikakis and Giovannucci, 2017) was developed by splitting the imaging field into overlapping patches and estimating the transformation for each pixel *via* upsampling. The computational speed is slow for these motion correction methods, thus some efforts for improving the speed have been made. For instance, Moco (Dubbs et al., 2016) was programmed in Java and achieved faster motion correction than TurboReg by implementing a discrete Fourier transform and cache-aware upsampling. Similarly, fast Fourier transform was applied for processing motion correction in the Suite2p toolbox (Pachitariu et al., 2017).

An alternative type of image registration approach is based on feature detection. These approaches perform by extracting features through a global intensity gradient such as DoG (difference of Gaussians). In this way, the feature detection approaches to extracting features refer to representative structures. Thus, feature detection-based algorithms, such as SIFT (Lowe, 2004), ORB (Rublee et al., 2011), and AKAZE (Alcantarilla et al., 2013), have been used for image registration. However, these approaches are also relatively computationally expensive and perform unsatisfactorily in image registration applications.

Since the rise of deep learning methods, which have achieved state-of-the-art results in many research fields, deep learning-based image registration algorithms have been rapidly developed and used in many image registration applications (Haskins et al., 2020). However, these methods have also encountered the issue of high computational power demand (Thompson et al., 2020), which may limit their applications in online imaging experiments.

The current image registration-based motion correction methods are either computationally expensive or difficult to implement with a conventional computer, and some suffer from both disadvantages. In this work, we focus on rigid motion correction. As suggested in previous research (Mitani and Komiyama, 2018), rigid transformation is quite efficient when imaging with resonant scanners; the high sampling rate can only induce negligible within-frame motion distortion. With the goal of improving both computational speed and accuracy, we propose a fast image feature extraction and registration (FIFER) method for motion correction of two-photon Ca^2+^ imaging data. Our proposed method is based on an image density estimation and clustering approach (Hinneburg and Keim, 1998), which is fast and robust in the extraction of imaging data features. The efficacy of the proposed motion correction method was first assessed with raw two-photon Ca^2+^ imaging data at both the neuronal population and single-spine scales. The quantitative evaluation of motion-corrected imaging data demonstrates that our method provides superior performance in comparison to other motion correction approaches, and thus provides a powerful solution to facilitate online functional imaging and photostimulation experiments in neuroscience research.

## Materials and Methods

### Data Acquisition

Adult (8–12 weeks old) male C57BL/6J mice were obtained from the Laboratory Animal Center at the Third Military Medical University. All experimental procedures related to the use of animals were approved by the Third Military Medical University Animal Care and Use Committee and were conducted in accordance with institutional animal welfare guidelines.

For two-photon imaging in head-fixed awake mice (Li et al., 2018), we first glued a titanium head post to the skull for head fixation under isoflurane anesthesia. Three days after surgery, animals received 1 ml of water supply per day for 2–3 days and then underwent training and testing sessions with water deprivation in their home cages. For imaging experiments with Cal-520 AM (Tada et al., 2014), we exposed the right primary auditory cortex of the mouse (Chen et al., 2011). Cal-520 AM was dissolved in DMSO with 20% Pluronic F-127 to a final concentration of 567 μM with artificial cerebral spinal fluid (ACSF) for bolus loading. With a standard pipette solution, the solution was diluted 1/10 for cell loading. After that, we used a micropipette filling with the solution and inserted coaxially into the targeted cortex. Finally, the dye-containing solution was ejected in the cortical area by applying a pressure pulse to the pipette. Ca^2+^ imaging was performed approximately 2 hours after dye injection and lasted for up to 8 hours (Stosiek et al., 2003). The mice were trained to perform a sound-evoked licking task (Wang et al., 2020).

The imaging experiment was conducted with a custom-built two-photon microscope system constructed with a 12 kHz resonant scanner (model “LotosScan 1.0,” Suzhou Institute of Biomedical Engineering and Technology, Suzhou, China), in accordance with the set-up described previously (Jia et al., 2010, 2014). Two-photon excitation light was delivered by a mode-locked Ti:Sa laser (model “Mai-Tai DeepSee,” Spectra Physics, CA, United States), and a 40 × /0.8 NA water-immersion objective (Nikon, Minato, Japan) was used for imaging. For Ca^2+^ imaging experiments, the excitation wavelength was set to 920 nm. For neuronal population imaging, images of 600 × 600 pixels were acquired at a 40 Hz frame rate. The size of the field-of-view was approximately 200 × 200 μm. The average power delivered to the brain was 30–120 mW. For dendritic spine imaging, we used the LOTOS procedure (Chen et al., 2011, 2012). The images, consisting of 250 × 250 pixels, were acquired at a 160 Hz frame rate. The size of the field-of-view was therefore reduced to approximately 25 × 25 μm. The average power delivered to the brain was 15–30 mW.

### Motion Correction Method

#### Motion Correction Pipeline for Two-Photon Imaging Data

**FIGURE 1:**
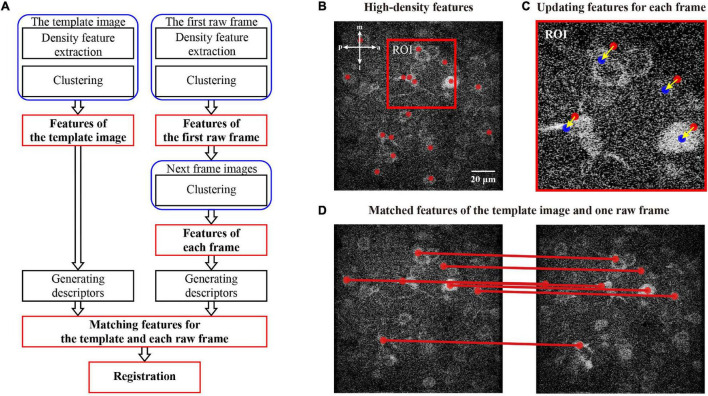
Processing pipeline for two-photon Ca^2+^ imaging data. **(A)** Flowchart of the proposed motion correction method. The red marked modules are the key steps in the processing flowchart. **(B)** Illustration of the feature (red dots) extraction for the imaging data. **(C)** Illustration of the feature update for a raw frame. The features of the current frame and the next frame are marked as the red and blue dots, respectively. **(D)** Illustration of matching features between template image and raw frame. The anatomical direction: a, anterior; m, medial; p, posterior; l, lateral.

Fast image feature extraction and registration (FIFER) operates in a rigid correction flow (Figure 1A). Initially, we generate features for an image with a density-based estimating and clustering algorithm (Figure 1B). This process begins a calculation with the template image (here we used the first frame of imaging data) and the first raw frame for correction (i.e., the second frame of imaging data). After obtaining features of the template and first raw frame, we then generated features for the rest of the raw frames by updating the features obtained from the first raw frame with a fast process (Figure 1C). After we obtained features for each frame, we matched the features between the template image and the raw frames according to the similarity metric (Figure 1D). Finally, we calculated the rigid transformation parameters and conducted the image registration. The computational details of each step are described in the following sections. In the figures, the brightness of the representative two-photon images was adjusted for better visualization.

#### Generating Features From Two-Photon Image

**FIGURE 2:**
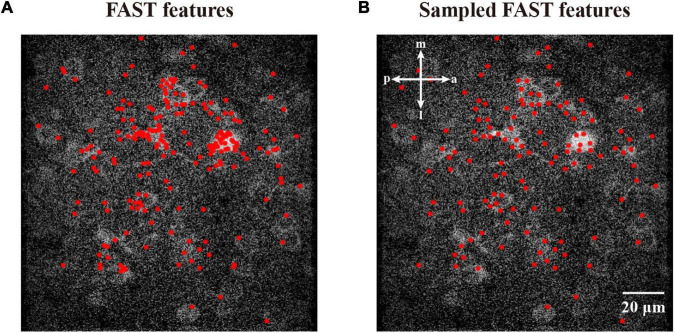
Features from accelerated segment test feature detection and sampled FAST features. **(A)** The corner features detected by FAST algorithm. **(B)** The sampled FAST features are uniformly distributed for reducing the computational cost. The features are represented as red dots.

**FIGURE 3:**
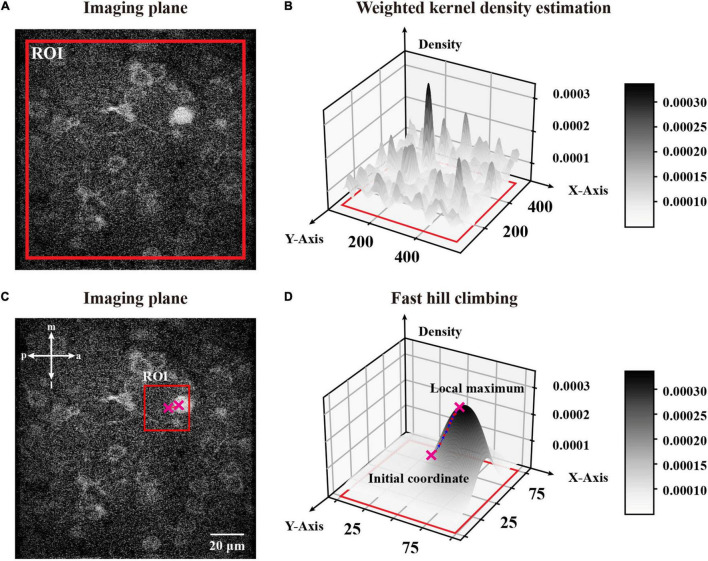
A global W-KDE for a two-photon image and a visual demonstration of fast hill climbing to update initial coordinates. **(A)** A representative two-photon image within a global range ROI marked in red. **(B)** A topography of global W-KDE. The density map indicated with the grayscale bar painted on the right. **(C)** An initial coordinate obtained from sampled FAST features with a 101 pixels-sized ROI centered by it. We mark the ROI in red and plot the initial coordinate and the local maximum with crosses that are also plotted in panel **(D)**. **(D)** A W-KDE of this ROI is to reveal the topography and process of fast hill climbing. The density map is indicated with the grayscale bar painted on the right. Each iteration of this feature is marked on the topography by pink dots.

To perform image density-based feature estimating and clustering, we first initialized the generation of features with the density estimate. To minimize the computational cost, we acquired the coordinates around the high-intensity area, instead of randomly inputting coordinates, to decrease the cost for the following hill climbing procedure. In this work, we assume that the features from accelerated segment test (FAST) (Rosten et al., 2008) detector is a suitable algorithm to detect corners with high speed. Figure 2A demonstrates that the corners detected by the FAST algorithm are mostly distributed in high-intensity areas. To reduce the number of repeated FAST features, we down-sampled these coordinates by merging those near each other (Figure 2B). Based on the initial FAST-detected features, we described the distribution of the empirical density field in an image. Inspired by Denclue (Hinneburg and Keim, 1998), we propose a weighted kernel density estimation (W-KDE) algorithm, with which we mapped each image pixel to a Gaussian kernel function weighted by this pixel’s image value as its estimated density:


(1) $$\begin{array}{c} {\hat{\text{f}}}_{Density}\left(\vec{p}\right)=\frac{1}{h^{2}\sum_{i=1}^{n}I\left({\vec{p}}_{i}\right)}\sum_{i=1}^{n}\text{I}\left({\vec{p}}_{i}\right)\cdot {\text{K}}_{Gaussian}\left(\frac{\vec{p}-{\vec{p}}_{i}}{h}\right)\\ {\text{K}}_{Gaussian}\left(\vec{u}\right)={\left(2\pi \right)}^{-1}\cdot e^{-\frac{{\left(\vec{u}\right)}^{2}}{2}} \end{array}$$

where $\vec{p}=\left\langle x,y\right\rangle$ is an arbitrary coordinate in a partitioned ROI (region of interest) of an image, ${\vec{p}}_{i}=\left\langle x_{i},y_{i}\right\rangle$ is one coordinate in the ROI, $\text{I}\left({\vec{p}}_{i}\right)$ is the pixel value in this position ${\vec{p}}_{i}$, *n* is the number of pixels sampled from the ROI, *h* is a bandwidth to smooth the density distribution, and ${\text{K}}_{Gaussian}\left(\vec{u}\right)$ is a Gaussian kernel function which maps a two-dimensional vector $\vec{u}$ onto a constant. Considering the distance between a given coordinate and pixel intensity, we describe the influence from one pixel ${\vec{p}}_{i}$ with a pixel value of $\text{I}\left({\vec{p}}_{i}\right)$ to an arbitrary coordinate $\vec{p}$ of the image as $\text{I}\left({\vec{p}}_{i}\right)\cdot {\text{K}}_{Gaussian}\left(\frac{\vec{p}-{\vec{p}}_{i}}{h}\right)$, which is quantified by a weighted Gaussian kernel function. Thus, we could estimate the density of an arbitrary coordinate $\vec{p}$ in a partitioned ROI by calculating the sampled ROI’s pixel component density and normalizing the sum of these components, which ensures that the whole distribution is a normalized density distribution. In brief, ${\hat{\text{f}}}_{Density}\left(\vec{p}\right)$ is the weighted mixture density of position $\vec{p}$ within the total influence of the ROI. To demonstrate that, we selected a fully sized ROI around an image and calculated each pixel’s density given the ROI (Figure 3A) through W-KDE and revealed the density field (Figure 3B) to visualize the density distribution.

Since we estimated a ROI’s density distribution, we analyzed its ridge topography for feature extraction. Here, we obtained the coordinates of high intensity regions and hence determined the local maximums as cluster centers (Hinneburg and Keim, 1998). The eq. (2) shows the derivation of W-KDE function with the hill climbing algorithm:


(2) $$\begin{array}{c} \nabla {\hat{\text{f}}}_{Density}\left(\vec{p}\right)=\frac{1}{h^{4}\sum_{i=1}^{n}\text{I}\left({\vec{p}}_{i}\right)}\sum_{i=1}^{n}\text{I}\left({\vec{p}}_{i}\right)\cdot {\text{K}}_{Gaussian}\left(\frac{\vec{p}-{\vec{p}}_{i}}{h}\right)\\ \;\cdot \left(\vec{p}-{\vec{p}}_{i}\right)\\ \;{\vec{p}}^{t+1}={\vec{p}}^{t}+\delta \frac{\nabla {\hat{\text{f}}}_{Density}\left({\vec{p}}^{t}\right)}{{||\nabla {\hat{\text{f}}}_{Density}\left({\vec{p}}^{t}\right)||}_{2}} \end{array}$$

where $\nabla {\hat{\text{f}}}_{Density}\left(\vec{p}\right)$ is the gradient of an arbitrary coordinate $\vec{p}$ in the ROI, and δ is the step stride in hill climbing. Other variables are the same as W-KDE equation. However, a fixed-step hill climbing algorithm is generally poor in efficiency. Therefore, we adapted a fast automatically step-adapted hill climbing approach in our algorithm, which was based on previous work (Hinneburg and Gabriel, 2007), as eq. (3) shows.


(3) $$\begin{array}{c} {\vec{p}}^{t+1}=\frac{\sum_{i=1}^{n}\text{I}\left({\vec{p}}_{i}\right)\cdot {\text{K}}_{Gaussain}\left(\frac{{\vec{p}}^{t}-{\vec{p}}_{i}}{h}\right)\cdot {\vec{p}}_{i}}{\sum_{i=1}^{n}\text{I}\left({\vec{p}}_{i}\right)\cdot {\text{K}}_{Gaussain}\left(\frac{{\vec{p}}^{t}-{\vec{p}}_{i}}{h}\right)}\\ \frac{{\hat{\text{f}}}_{Density}\left({\vec{p}}^{t+1}\right)-{\hat{\text{f}}}_{Density}\left({\vec{p}}^{t}\right)}{{\hat{\text{f}}}_{Density}\left({\vec{p}}^{t+1}\right)}\leq \varepsilon \end{array}$$

where ε is a given restriction to end hill climbing that also represents the precision of the local maximum. Therefore, we optimized the hill climbing to a faster algorithm by reducing the iteration numbers based on the increase in rate of density. Alternatively, we can simply stop the iteration when a coordinate update distance is smaller than a threshold η.


(4) $${||{\vec{p}}^{t+1}-{\vec{p}}^{t}||}_{2}\leq \eta$$

**FIGURE 4:**
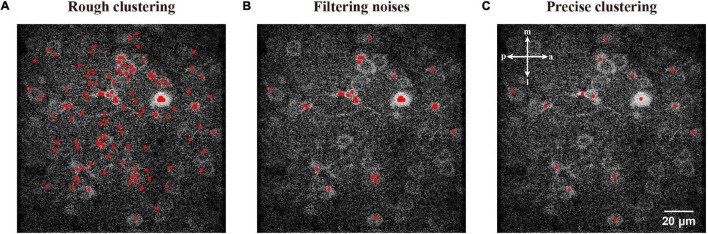
Demonstration of the processing used to generate precise high-density features. **(A)** The sampled FAST features were processed by rough clustering. **(B)** A filtering of features to reduce noises. **(C)** Clustering with a precise termination and merging the coincident features.

Hence we can start with any coordinate in the ROI and cluster it to a local maximum, which is the top of a ridge topography and probably represents a neuronal object. To update the initial coordinates to a local maximum, we used a ROI centered by the feature with an appropriate size of 101 pixels (Figure 3C). After repeating the fast hill climbing procedure (Figure 3D) to the coordinates of sampled FAST features with a rough termination, where we calculate step distance of each feature during iterations and simply terminate the fast hill climbing when the step distance is smaller than a rough constant η in eq. (4), and we obtained rough features with noise (Figure 4A). Then, we sorted all of the rough features by their estimated densities and treated the upper quartile features as filtered results (Figure 4B). Finally, we repeated the fast hill climbing procedure on rough features with a precise termination, where we finally terminate the hill climbing when the density change rate of each feature is smaller than a critical value as described in eq. (3), and merged the coincident features (Figure 4C). After conducting these processes on the template image and the first raw frame individually, we obtained the initial pair of image features.

#### Updating Features With an Efficient Mode

**FIGURE 5:**
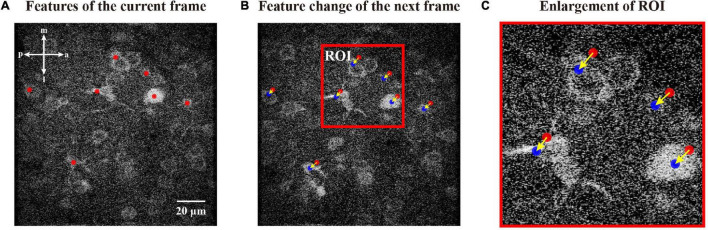
Updating features in a consecutive mode. **(A)** Features of current frame marked by red dots. **(B)** Features of the next frame updated from features of the current frame. We indicate features of the next frame with blue dots and those of the current frame with red dots. **(C)** Enlargement of the respective ROI in panel **(B)**, i.e., the change flow of coordinates during this iteration. The arrows indicate the movement directions.

Starting the calculation for the features of the first raw frame, we updated the features of the current frame to those of the next frame through the following procedure (Figure 5). First, we used a fixed-size ROI centered on every coordinate of the feature in the current frame under the hypothesis that the features between two consecutive frames are adjacent. Then, we applied the fast hill climbing algorithm to achieve local maxima in the same way as before. This procedure is ultrafast and efficient for updating features from consecutive imaging frames due to: (1) Only a small number of features, which were extracted from the first raw frame, were updated in this process; (2) features between two consecutive frames were normally adjacent, so the hill climbing iteration was stopped quickly; and (3) the fast hill climbing algorithm could automatically adapt the step of hill climbing depending on the distance to the local maximum. Therefore, our approach has an advantage in computational efficiency by updating features from consecutive imaging frames.

#### Matching Corresponding Features

**FIGURE 6:**
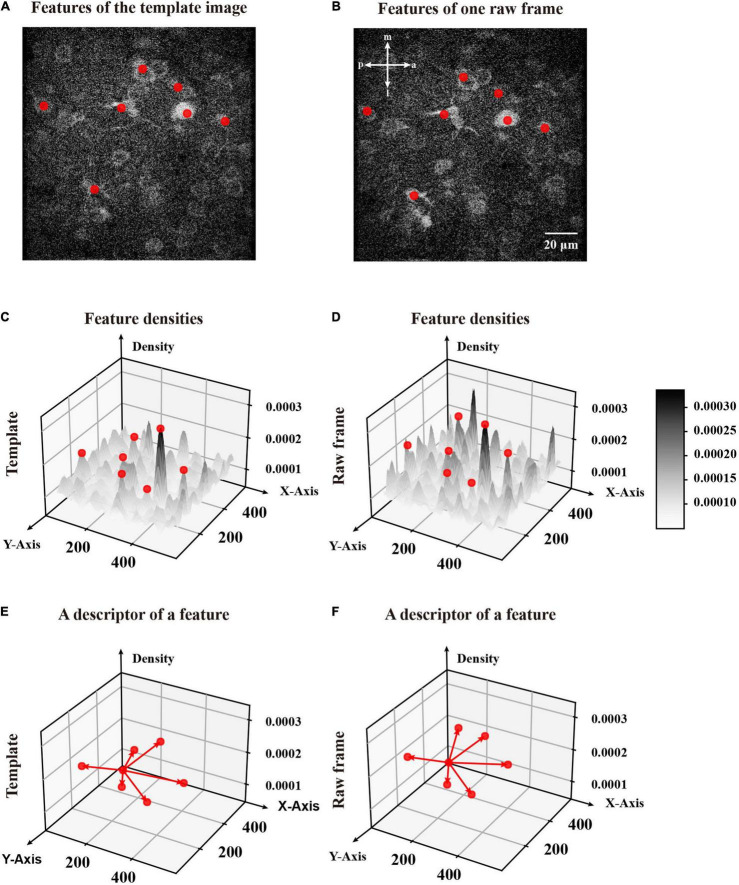
A visualization of the process to generate the descriptor. **(A)** The features for the template image obtained by FIFER features are marked by red dots. **(B)** A raw frame to be corrected with its corresponding features. **(C)** The density distribution of the template image with corresponding features. **(D)** The density distribution of the raw frame with corresponding features. The density maps in panels **(C)** and **(D)** are indicated with the grayscale bar painted on the right. **(E)** The descriptor of a feature selected from the template image. **(F)** The descriptor of a feature from the raw frame. This descriptor represents a collection of the differences between itself and all the other features in panels **(E,F)**.

**FIGURE 7:**
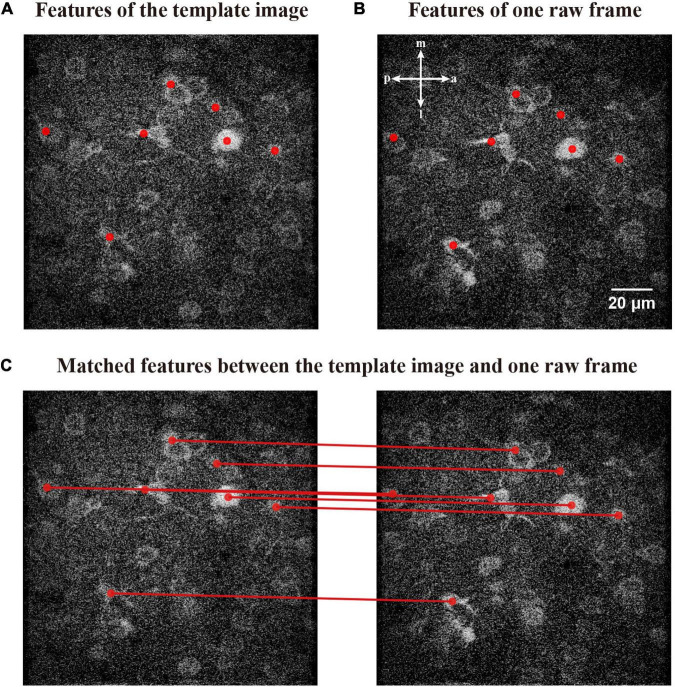
Matching relationships between the features of template image and raw frame. **(A)** The features obtained by FIFER of the template image; features are marked by red dots. **(B)** A raw frame to be corrected with its corresponding features. **(C)** The corresponding coupling of the features; the matched relationships are indicated by red lines.

When we obtained features of the template image (Figure 6A) and one raw frame in the imaging sequence (Figure 6B), we matched those features to obtain transformation parameters for image registration. We then constructed the feature vector in three dimensions: the *x*- and *y*-axis positions and the normalized density (Figures 6C,D). In addition, we assumed that the template image and the raw frame have similar image structure in terms of position and density. Based on this hypothesis, we deduced that the coupled features in the template image and raw frame have the same relative position and estimated density. Thus, we propose to create a descriptor for each feature by reserving the differences between this feature and the rest (Figures 6E,F).


(5) $$\begin{array}{c} \left\{ \begin{array}{c} P_{Template}=\left[{\vec{p}}_{1}^{Temp},{\vec{p}}_{2}^{Temp}\ldots {\vec{p}}_{k_{1}-1}^{Temp},{\vec{p}}_{k_{1}}^{Temp}\right]\\ P_{Target}=\left[{\vec{p}}_{1}^{Targ},{\vec{p}}_{2}^{Targ}\ldots {\vec{p}}_{k_{2}-1}^{Targ},{\vec{p}}_{k_{2}}^{Targ}\right] \end{array}\right.\\ \left\{ \begin{array}{c} \begin{array}{c} Descriptor\left({\vec{p}}_{\alpha }^{Temp}\right)=\left[{\vec{u}}_{1},{\vec{u}}_{2},,{\vec{u}}_{k_{1}-1},{\vec{u}}_{k_{1}}\right]\\ Descriptor\left({\vec{p}}_{\beta }^{Targ}\right)=\left[{\vec{v}}_{1},{\vec{v}}_{2},\ldots ,{\vec{v}}_{k_{2}-1},{\vec{v}}_{k_{2}}\right] \end{array}\\ \begin{array}{c} {\vec{u}}_{k_{1}}={\vec{p}}_{k_{1}}^{Temp}-{\vec{p}}_{\alpha }^{Temp}\\ {\vec{v}}_{k_{2}}={\vec{p}}_{k_{2}}^{Targ}-{\vec{p}}_{\beta }^{Targ} \end{array} \end{array}\right. \end{array}$$

where the descriptors ${\vec{p}}_{\alpha }^{Temp}$ or ${\vec{p}}_{\beta }^{Targ}$ are in their groups of point sets. After obtaining the descriptor of each feature, we matched the features by comparing the similarity of their descriptors. To match two feature descriptors, the corresponding elements between two descriptors were calculated for the Jaccard similarity coefficient with a parameter ζ_1_ set as the tolerable limit. If the calculated Jaccard similarity coefficient was larger than a given threshold ζ_2_, their corresponding two features were successfully matched. Eq. (6) describes this matching process.


(6) $$\left\{ \begin{array}{c} J\left(Descriptor\left({\vec{p}}_{\alpha }^{Temp}\right),Descriptor\left({\vec{p}}_{\beta }^{Targ}\right)\right)\\ =\frac{Descriptor\left({\vec{p}}_{\alpha }^{Temp}\right)\cap Descriptor\left({\vec{p}}_{\beta }^{Targ}\right)}{Descriptor\left({\vec{p}}_{\alpha }^{Temp}\right)\cup Descriptor\left({\vec{p}}_{\beta }^{Targ}\right)}\\ =\frac{\tau }{2\left(k_{1}+k_{2}-\tau \right)}\\ \tau =\sum_{m,n=1}^{k_{1},k_{2}}L\left(\frac{2{||{\vec{u}}_{m}-{\vec{v}}_{n}||}_{2}}{{||{\vec{u}}_{m}||}_{2}+{||{\vec{v}}_{n}||}_{2}}\right)-\tau_{same}\\ L\left(\sigma \right)=\left\{ \begin{array}{c} 1,\sigma \leq \zeta_{1}\\ 0,\sigma >\zeta_{1} \end{array}\right. \end{array}\right.$$

where τ_*same*_ represents the counts of multi-matching features. We demonstrate matching the features of a template image (Figure 7A) with a raw frame (Figure 7B). The matched relations of those features are shown in Figure 7C.

**FIGURE 8:**
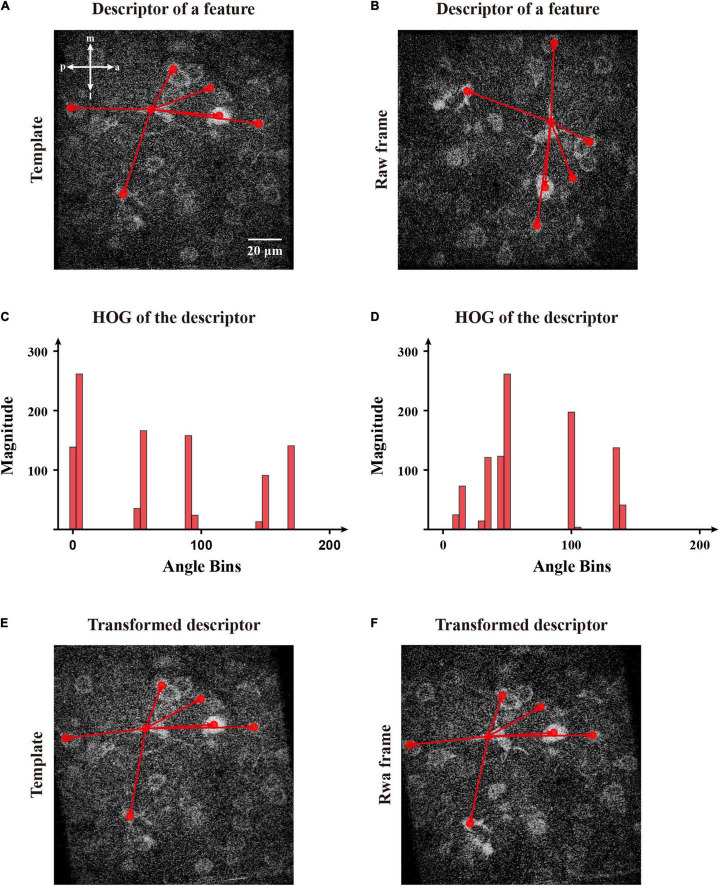
The calculation of direction of the selected features and transformation of corresponding descriptors for matching. **(A)** The features obtained by FIFER; features are marked by red dots. **(B)** A raw frame with a rotation deviation and its corresponding features. The red arrows represent a two-dimensional descriptor of the selected feature in panels **(A,B)**. **(C)** The histogram of magnitude in directions bins of the template feature’s descriptor. **(D)** The histogram of magnitude in directions bins of the raw frame feature’s descriptor. **(E)** The descriptor of the selected template feature within its main direction. **(F)** The transformed descriptor of the selected frame feature within its main direction.

In addition, we calculated the rotation transformation parameters for the image feature descriptors. Inspired by SIFT (Lowe, 2004), we used a histogram of oriented gradients (HoG) to set a constructive main direction to transform each feature for matching images (Figures 8A,B). Initially, we created several bins to calculate the distributions of the summed magnitude of vectors within 36 directions for each descriptor. Then, we decomposed each element of a descriptor of magnitudes into the upper and lower boundaries and counted the total magnitude in those 36 directions (Figures 8C,D). After that, we determined the main direction whose bin had the largest total magnitude. To obtain continuous directions, we used parabola interpolation to estimate a relative precise direction. Since we obtained the individual direction of a descriptor, we transformed the descriptor for matching features (Figures 8E,F). Then, we estimated a rotation angle by applying singular value decomposition (SVD) to multiple couples of the matched features (Besl and McKay, 1992). Hence we calculated the covariance matrix among two groups of features and applied SVD to obtain a transformation matrix and perform the registration of the image.

### Evaluation of Motion Correction Performance

For the acquired two-photon imaging datasets, the similarities between the template image and corrected imaging data frames were calculated to evaluate motion correction performance. Here, four popular image quality metrics, including the normalized root mean square error (NRMSE), the peak signal-to-noise ratio (PSNR), the structural similarity (SSIM) index, and the normalized mutual information (NMI), were adopted in our evaluation tests. A smaller NRMSE value and a larger PSNR/SSIM/NMI value indicate better image correction quality (Luo et al., 2021). The calculation time was measured for processing each raw two-photon image of 600 × 600, 512 × 512 (neuronal population imaging) or 250 × 250 pixels (dendritic spine imaging) using an AMD Ryzen 7 5800X 3.8 GHz CPU, with 32 GB RAM.

## Results

### Validation of Motion Correction by Fast Image Feature Extraction and Registration

**FIGURE 9:**
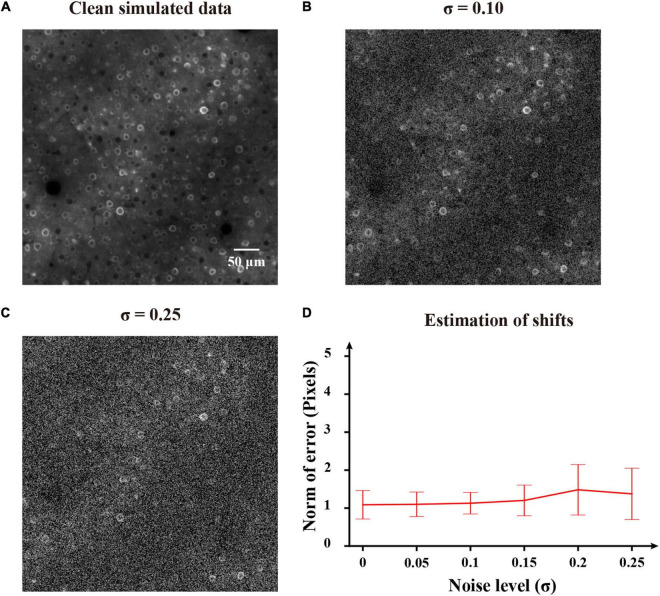
Application of FIFER to simulated data. **(A)** Template image (the first frame) of the clean simulated data. **(B)** Template image (the first frame) of the simulated data added with Gaussian noise of a standard deviation σ = 0.10. **(C)** Template image (the first frame) of the simulated data combined with Gaussian noise of a standard deviation σ = 0.25. **(D)** Estimation of shifts for the simulated data of different noise levels. FIFER’s error is calculated as the 2d-norm between the estimated offsets by FIFER and ground truth offsets (artificially introduced).

To first perform an experimental validation of the proposed method, we evaluated the motion correction results on simulated imaging data. Here we used the NAOMi simulation toolbox (Song et al., 2021) to simulate two-photon imaging data. We set the numerical aperture (NA) of the objective lens as 0.8, the NA of the illumination light as 0.6 and the laser power as 40 mW. The generated data video had a size of 500 × 500 pixels and 100 frames. Figure 9A shows a representative example of simulated clean two-photon image. Then the synthetic noisy imaging data were generated with random spatial shifts and added Gaussian noise as the previous work (Pnevmatikakis and Giovannucci, 2017). Gaussian noise with zero mean and standard deviation sampled from [0, 0.25] were added to the clean two-photon images to generate testing images. Figures 9B,C show two noisy shifted images (noise levels: σ = 0.10 and σ = 0.25). As we can see (Figure 9D), the FIFER algorithm can estimate the shifts remarkably well. We evaluated the performance of motion correction for the simulated data with different noise levels, and the estimate errors do not change much with the noise level increases. Therefore, our proposed method achieved good performance on the simulation data test.

**FIGURE 10:**
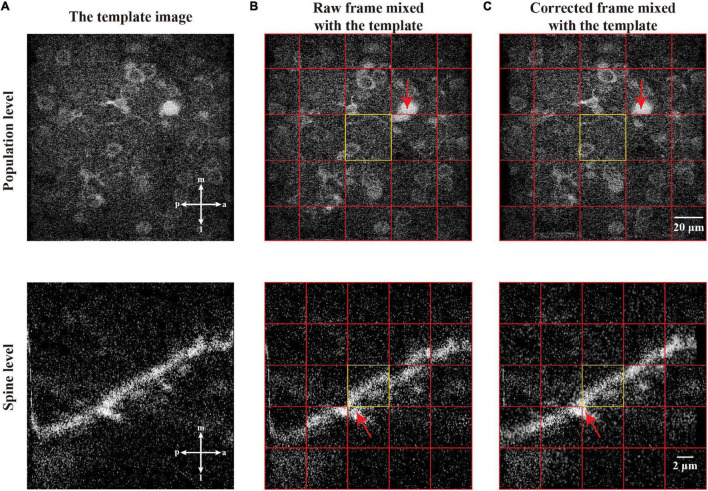
The motion correction effect in single raw frame of two-photon imaging data. **(A)** The template images of the population level and spine level. **(B)** Raw frames from population level and spine level with motion artifacts which are mixed with corresponding template image. **(C)** The correction of the raw frames, which are mixed with corresponding template image. In a mixed image, the image patches from the template and single frame are equally distributed and marked by red grid lines. The equally distribution means any two adjacent patches are from the template image and single frame, respectively. Each center of the mixed image is a patch from corresponding template image, which is marked by yellow rectangle. The red arrows indicate the performance of registration.

To further evaluate the performance of motion correction, we used two different two-photon imaging scales of datasets, i.e., the scale of a neuronal population (Figure 10, top) and the scale of a dendritic spine (Figure 10, bottom). Figures 10A,B shows the examples of the template images and the raw imaging frames (mixed with template images) to be corrected, respectively. After we applied our motion correction method for processing the raw imaging frames, the two representative frames were aligned to their corresponding templates (Figure 10C). This figure demonstrates that the raw frames were correctly transformed (here, a translation) to be matched with the template images.

**FIGURE 11:**
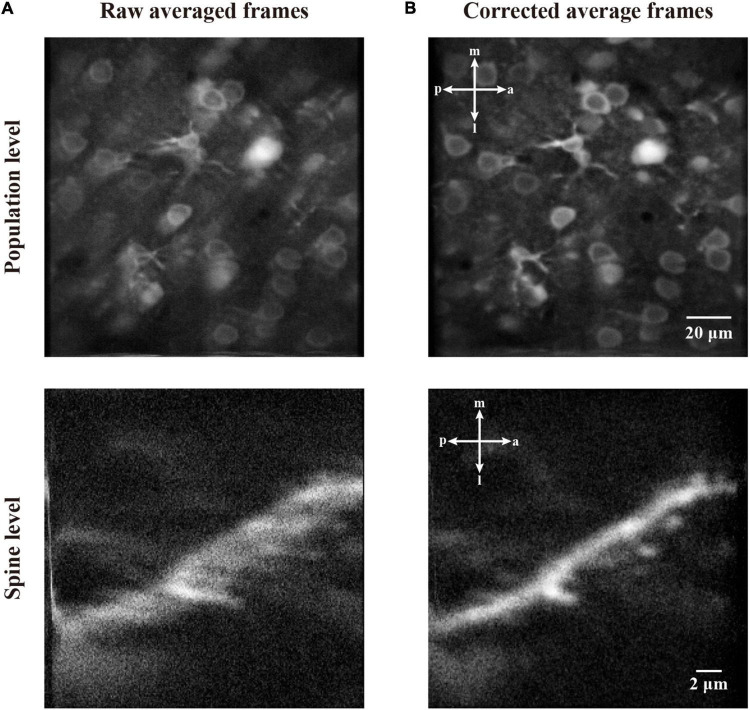
The correction effect in average frame of two-photon imaging data. **(A)** The average of raw frames at population level and spine level. **(B)** The average of motion corrected frames at population level and spine level.

In Figure 11, we demonstrate the motion correction effects by showing the average image of the imaging data before and after motion correction. As the motion artifacts distort some of the imaging frame sequences, the average of a functional imaging data might suffer from inconsistency. As can be seen from the averaged raw image of the two imaging datasets (Figure 11A), both the neuronal population and dendritic spine imaging data show a blur effect due to motion artifacts during recording. By contrast, after we performed our motion correction method with the raw imaging data, the average of corrected images shows greatly improved quality, and the cells and spines are clearly visible without obvious blurred effect (Figure 11B). Thus, the imaging data were successfully restored.

**FIGURE 12:**
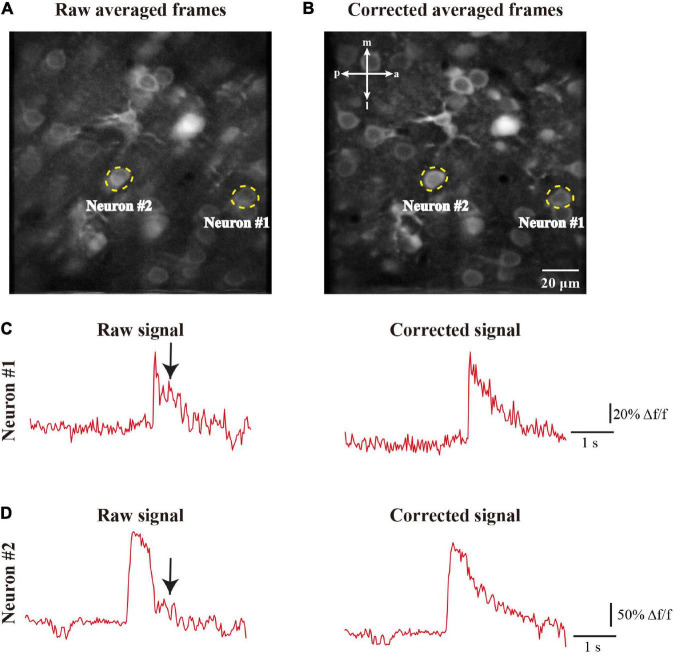
The motion correction effect of the neuronal signals. **(A)** Two representative neurons in the raw averaged frames, the neurons are indicated by dashed circles in yellow. **(B)** Two representative neurons in the corrected averaged frames. **(C)** The raw and corrected Ca^2+^ signals of neuron #1. **(D)** The raw and corrected Ca^2+^ signals of neuron #2. The black arrows indicate the distorted period due to motion artifacts.

We further addressed the effects of motion corrected neuronal activity as a time series of fluorescence changes. We identified the individual neurons and extracted their Ca^2+^ signals from the imaging frames to assess the change dynamics of Ca^2+^ transients both before (Figure 12A) and after (Figure 12B) motion correction. The raw imaging data exhibit that the motion resulted in spike-like changes (Figure 12C, left) or even larger distortions (Figure 12D, left) in the time series, which are indicated by the black arrows in Figures 12C,D. After we processed the raw imaging data with our method, the motion artifact-induced changes were successfully reduced and the neuronal signals were clearly restored (Figures 12C,D, right). Hence, the quality of individual Ca^2+^ transients processed by motion correction algorithm was clearly higher than that of the raw signals, which can facilitate downstream analyses, e.g., the detection of soma (Guan et al., 2018) and neuronal Ca^2+^ transients.

### Comparison of Different Methods for Motion Correction

To further assess the motion correction performance of our proposed method, we compared FIFER with other popular image registration methods. We tested them with three two-photon imaging datasets, including neuronal population (*n* = 200 and *n* = 1825) and dendritic spine (*n* = 1500) imaging frames to evaluate the motion correction performance of the methods. The image registration methods used for comparison consisted of two groups: feature-based methods and intensity-based methods. For the classic feature-based methods, the SIFT (Lowe, 2004), ORB (Rublee et al., 2011), and AKAZE (Alcantarilla et al., 2013) methods were used for comparison. For the widely used intensity-based methods, TurboReg (Thevenaz et al., 1998), Moco (Dubbs et al., 2016), NoRMCorre (Pnevmatikakis and Giovannucci, 2017), the real-time processing method (Mitani and Komiyama, 2018), and Suite2p (Pachitariu et al., 2017) were used for testing.

First, we tested FIFER and the abovementioned methods with our dataset of neuronal population imaging. As the testing results showing in Table 1, our FIFER method exhibits superior motion correction performance compared to existing image registration approaches in terms of NRMSE (0.9131 ± 0.0416), PSNR (19.7529 ± 0.3930), SSIM (0.1972 ± 0.0105), and NMI (0.0281 ± 0.0027). In addition, the calculation time of our proposed method is just 2.92 ms for processing each image. For the feature-based methods, SIFT shows better relative motion correction performance than ORB and AKAZE with a longer computational time, and ORB is relatively fast but has poor correction ability. For the intensity-based methods, we tested TurboReg with two working modes: Accurate and Fast. The Accurate mode of TurboReg achieved a better correction performance than that of Fast mode, while also requiring a longer calculation time. As the previous work reported, Moco achieved a fast processing of the data, using 26.92 ms for processing each frame. However, the motion correction performance of Moco is just comparable to the Fast mode of TurboReg. Moreover, we also tested NoRMCorre with its two modes, Rigid and Non-rigid. The Non-rigid mode of NoRMCorre performed well and was the closest to matching the motion correction performance achieved by FIFER, ranking second in our comparative analysis. However, the calculation time for the Non-rigid mode was the longest among the tested methods. In comparison, the Rigid mode of NoRMCorre also achieved good results with a much shorter time for processing. For the real-time processing method, it was very fast when processing the raw frames, requiring 3.85 ms for each frame, and its correction performance was as the same level as that of Moco and the Fast mode of TurboReg. For correcting the images with Suite2p, it used 11.91 ms for processing each frame and revealed similar performance as that of Moco. Hence Suite2p achieved a moderate motion correction accuracy and speed. Taken together, our proposed method not only provides superior motion correction results (*P* < 0.05, paired *t*-test), but also uses minimum processing time compared to the other tested methods.

**TABLE 1:** Comparison of fast image feature extraction and registration (FIFER) with other methods (Mean ± SD) for neuronal population imaging dataset (*n* = 200 frames, 600 × 600 pixels for each frame).

Method	Metric (*P*-value: FIFER vs. other method)				Time (ms)
	NRMSE	PSNR	SSIM	NMI	
SIFT	0.9922 ± 0.0641 (*P* = 1.12e-35)	19.0407 ± 0.5627 (*P* = 6.13e-36)	0.1726 ± 0.0224 (*P* = 2.46e-35)	0.0217 ± 0.0059 (*P* = 7.30e-35)	105.23
ORB	1.0202 ± 0.0566 (*P* = 2.91e-57)	18.7942 ± 0.4903 (*P* = 2.00e-57)	0.1594 ± 0.0248 (*P* = 1.42e-51)	0.0191 ± 0.0065 (*P* = 3.15e-45)	26.87
AKAZE	1.0082 ± 0.0703 (*P* = 8.81e-45)	18.9045 ± 0.6131 (*P* = 6.41e-45)	0.1740 ± 0.0181 (*P* = 1.10e-41)	0.0223 ± 0.0045 (*P* = 2.22e-41)	52.41
TurboReg (Accurate)	0.9738 ± 0.0345 (*P* = 2.59e-42)	19.1906 ± 0.3063 (*P* = 1.93e-42)	0.1799 ± 0.0124 (*P* = 1.68e-55)	0.0236 ± 0.0012 (*P* = 2.78e-69)	140.87
TurboReg (Fast)	1.0369 ± 0.0212 (*P* = 2.46e-96)	18.6414 ± 0.1759 (*P* = 8.20e-94)	0.1700 ± 0.0110 (*P* = 4.53e-92)	0.0223 ± 0.0009 (*P* = 1.23e-85)	118.15
Moco	1.0361 ± 0.0211 (*P* = 9.23e-96)	18.6484 ± 0.1749 (*P* = 2.96e-93)	0.1715 ± 0.0109 (*P* = 3.41e-86)	0.0224 ± 0.0009 (*P* = 6.30e-84)	26.92
NoRMCorre (Rigid)	0.9601 ± 0.0422 (*P* = 9.29e-23)	19.3160 ± 0.3796 (*P* = 4.60e-23)	0.1842 ± 0.0119 (*P* = 2.17e-34)	0.0240 ± 0.0014 (*P* = 1.46e-56)	61.28
NoRMCorre (Non-rigid)	0.9521 ± 0.0181 (*P* = 1.46e-28)	19.3817 ± 0.1641 (*P* = 5.53e-29)	0.1922 ± 0.0090 (*P* = 1.28e-12)	0.0246 ± 0.0009 (*P* = 6.73e-53)	210.21
Real-time processing	1.0147 ± 0.0667 (*P* = 7.10e-49)	18.8815 ± 1.2811 (*P* = 4.70e-18)	0.1755 ± 0.0591 (*P* = 2.93e-7)	0.0254 ± 0.0270 (*P* = 1.55e-1)	3.85
Suite2p	1.0087 ± 0.0254 (*P* = 3.99e-73)	18.8821 ± 0.2166 (*P* = 3.11e-72)	0.1694 ± 0.0130 (*P* = 5.86e-82)	0.0226 ± 0.0013 (*P* = 1.06e-81)	11.91
**FIFER**	**0.9131 ± 0.0416**	**19.7529 ± 0.3930**	**0.1972 ± 0.0105**	**0.0281 ± 0.0027**	**2.92**

*The calculation time is for each frame. Statistical tests were calculated using the paired t-test.*

We next compared FIFER with those methods for another dendritic spine imaging dataset, because this is also a popular imaging scale. As the results (Table 2) demonstrate, our proposed method also provided the best correction results (*P* < 0.05, paired *t*-test) in terms of NRMSE (0.8490 ± 0.0508), PSNR (18.5548 ± 0.5045), SSIM (0.1791 ± 0.0149), NMI (0.0733 ± 0.0043), and the fastest processing speed (0.59 ms for processing each frame). Among the other tested methods, the Accurate mode of TurboReg achieved the second-best correction result, and the real-time processing method achieved the second fastest processing speed (0.76 ms for processing each frame).

**TABLE 2:** Comparison of FIFER with other methods (mean ± SD) for dendritic spine imaging dataset (*n* = 1500 frames, 250 × 250 pixels for each frame).

Method	Metric (*P*-value: FIFER vs. other method)				Time (ms)
	NRMSE	PSNR	SSIM	NMI	
SIFT	0.9253 ± 0.0815 (*P* = 7.06e-260)	17.8256 ± 0.7608 (*P* = 9.59e-264)	0.1657 ± 0.0192 (*P* = 2.78e-108)	0.0661 ± 0.0089 (*P* = 1.50e-254)	15.79
ORB	0.9344 ± 0.0783 (*P* = 1.11e-313)	17.7370 ± 0.7262 (*P* = 2.47e-319)	0.1630 ± 0.0191 (*P* = 2.32e-145)	0.0647 ± 0.0090 (*P* = 6.61e-295)	10.92
AKAZE	0.9312 ± 0.0809 (*P* = 1.90e-294)	17.7694 ± 0.7513 (*P* = 2.95e-299)	0.1628 ± 0.0202 (*P* = 7.10e-49)	0.0668 ± 0.0077 (*P* = 3.73e-280)	7.78
TurboReg (Accurate)	0.9064 ± 0.0292 (*P* = 1.45e-258)	17.9752 ± 0.2775 (*P* = 1.59e-264)	0.1539 ± 0.0090 (*P* = 9.99e-140)	0.0719 ± 0.0027 (*P* = 2.27e-76)	109.58
TurboReg (Fast)	0.9654 ± 0.0135 (*P* = 0)	17.4240 ± 0.1197 (*P* = 0)	0.1391 ± 0.0045 (*P* = 0)	0.0711 ± 0.0025 (*P* = 2.70e-154)	104.46
Moco	0.9604 ± 0.0111 (*P* = 0)	17.4687 ± 0.1000 (*P* = 0)	0.1411 ± 0.0043 (*P* = 0)	0.0715 ± 0.0025 (*P* = 1.80e-104)	9.24
NoRMCorre (Rigid)	0.9325 ± 0.0189 (*P* = 0)	17.7263 ± 0.1742 (*P* = 0)	0.1462 ± 0.0054 (*P* = 0)	0.0711 ± 0.0025 (*P* = 9.10e-175)	7.59
NoRMCorre (Non-rigid)	0.9301 ± 0.0147 (*P* = 0)	17.7480 ± 0.1361 (*P* = 0)	0.1485 ± 0.0049 (*P* = 0)	0.0720 ± 0.0026 (*P* = 5.10e-77)	57.4
Real-time processing	0.9550 ± 0.0254 (*P* = 0)	17.5269 ± 0.5620 (*P* = 0)	0.1417 ± 0.0222 (*P* = 0)	0.0718 ± 0.0096 (*P* = 5.54e-9)	0.76
Suite2p	0.9506 ± 0.0252 (*P* = 0)	17.5606 ± 0.2262 (*P* = 0)	0.1428 ± 0.0061 (*P* = 0)	0.0697 ± 0.0029 (*P* = 4.00e-323)	1.90
**FIFER**	**0.8490 ± 0.0508**	**18.5548 ± 0.5045**	**0.1791 ± 0.0149**	**0.0733 ± 0.0043**	**0.59**

*The calculation time is for each frame. Statistical tests were calculated using the paired t-test.*

To supplement the validation datasets, we further tested FIFER and those methods with a publicly available dataset provided by the CaImAn project (Giovannucci et al., 2019). The testing results (Table 3) show that, FIFER (1.77 ms for processing each frame) was clearly faster than all other algorithms, meanwhile it also achieved the best (*P* < 0.05, paired *t*-test) correction performance in terms of NRMSE (0.7930 ± 0.1346), PSNR (27.0004 ± 1.3278), SSIM (0.4632 ± 0.0382), and NMI (0.0434 ± 0.0033). These validation results indicate a high generalization ability of FIFER, as it performed consistently fast and accurate for processing multiple datasets acquired from different labs.

**TABLE 3:** Comparison of FIFER with other methods (Mean ± SD) for neuronal population imaging dataset (the CaImAn dataset file images_N.01.01, *n* = 1825 frames, 512 × 512 pixels for each frame).

Method	Metric (*P*-value: FIFER vs. other method)				Time (ms)
	NRMSE	PSNR	SSIM	NMI	
SIFT	0.9611 ± 0.1576 (*P* = 0)	25.3388 ± 1.4258 (*P* = 0)	0.3193 ± 0.1376 (*P* = 3.48e-295)	0.0218 ± 0.0161 (*P* = 0)	67.54
ORB	0.9523 ± 0.1563 (*P* = 0)	25.4124 ± 1.3676 (*P* = 0)	0.3744 ± 0.0696 (*P* = 0)	0.0283 ± 0.0097 (*P* = 0)	18.31
AKAZE	0.8843 ± 0.1436 (*P* = 0)	26.0484 ± 1.2999 (*P* = 0)	0.4022 ± 0.0433 (*P* = 0)	0.0346 ± 0.0039 (*P* = 0)	40.28
TurboReg (Accurate)	0.8789 ± 0.1365 (*P* = 0)	26.0914 ± 1.2283 (*P* = 0)	0.4093 ± 0.0367 (*P* = 0)	0.0347 ± 0.0025 (*P* = 0)	112.07
TurboReg (Fast)	0.8943 ± 0.1398 (*P* = 0)	25.9418 ± 1.2370 (*P* = 0)	0.4005 ± 0.0383 (*P* = 0)	0.0337 ± 0.0024 (*P* = 0)	109.20
Moco	0.8945 ± 0.1402 (*P* = 0)	25.9403 ± 1.2400 (*P* = 0)	0.4003 ± 0.0386 (*P* = 0)	0.0337 ± 0.0024 (*P* = 0)	15.00
NoRMCorre (Rigid)	0.8899 ± 0.1396 (*P* = 0)	25.9848 ± 1.2412 (*P* = 0)	0.4027 ± 0.0383 (*P* = 0)	0.0340 ± 0.0024 (*P* = 0)	39.77
NoRMCorre (Non-rigid)	0.8821 ± 0.1392 (*P* = 0)	26.0631 ± 1.2486 (*P* = 0)	0.4090 ± 0.0379 (*P* = 0)	0.0346 ± 0.0024 (*P* = 0)	264.38
Real-time processing	0.8897 ± 0.1390 (*P* = 0)	25.9905 ± 1.2984 (*P* = 0)	0.3976 ± 0.0401 (*P* = 0)	0.0342 ± 0.0119 (*P* = 1.70e-179)	2.70
Suite2p	0.8881 ± 0.1400 (*P* = 0)	26.0037 ± 1.2457 (*P* = 0)	0.4041 ± 0.0385 (*P* = 0)	0.0341 ± 0.0024 (*P* = 0)	8.28
**FIFER**	**0.7930 ± 0.1346**	**27.0004 ± 1.3278**	**0.4632 ± 0.0382**	**0.0434 ± 0.0033**	**1.77**

*The calculation time is for each frame. Statistical tests were calculated using the paired t-test.*

## Discussion

In this work, we proposed the FIFER method to perform motion correction for two-photon Ca^2+^ imaging data by extracting features *via* image density-based estimation and raw image registration. Using a regular personal computer, the proposed method showed promising performance in both computational speed and motion correction precision. The testing results showed that our method achieved strong results for not only simulated imaging data (Figure 9) but also both neuronal population and dendritic spine imaging datasets (Figures 10, 11). In addition, we demonstrated that the individual neuronal signal quality was clearly improved using our approach (Figure 12). The comparative analyses of FIFER against previously reported image registration approaches for motion correction in three imaging datasets (Tables 1–3) highlight the superior ability of our proposed method, as it is faster than other approaches while also achieving the best image correction accuracy. Hence, adopting our image correction method for online two-photon imaging experiments will benefit both functional imaging and closed loop photostimulation. The code implementing FIFER is published on the project’s GitHub page.

To extract features for matching images, we proposed a new algorithm, namely, W-KDE and density-based clustering, to extract the density features from a single frame of two-photon imaging data. Our proposed method outperformed the other feature-based and intensity-based methods for motion correction because it operates in a robust way to extract features, and then finds the optimal template matching solution. As our proposed method registered a pair of images by extracting local features, it reduced the differential contents from the global image. By contrast, the other feature-based methods, including SIFT, ORB, and AKAZE, did not show good performance. The reason for this might be that their feature extraction suffered from the signal-to-noise ratio issues with the imaging data. The insufficient common neuronal morphology or uneven background might cause classical feature-based motion correction methods to fail. Specially, the features extracted by our approach mostly represent some certain cellular structures: the feature marks the strongest Ca^2+^ signals of the soma or the dendritic spine, hence the extracted features by our approach are more accurate than the traditional feature extraction approaches for two-photon images. In addition, the intensity-based methods, including TurboReg, Moco, NoRMCorre, Suite2p and the real-time processing method, also perform image registration based on global image information. Hence, they might also encounter the issue that the intensity difference between the non-common areas (e.g., the noisy background) from the image pair may have a negative impact on the image registration and result in a sub-optimal image registration solution (Li et al., 2020). Furthermore, the sparse features extracted from foreground cellular structures by our approach hardly suffer from the background noise and might lead to an optimal solution. For instance, the dendritic spine imaging data only presented small common areas, so FIFER using local features achieved better results than other methods (Table 2). Therefore, the testing results show that our feature extraction and matching method provides robust results for correcting motion artifacts at different two-photon imaging scales.

Moreover, the testing results show that our method is ultrafast to find an optimal matching solution for an image pair. Only the real-time processing method achieved comparable calculation speed, and the other methods are much slower (Tables 1–3). With our algorithm, once features were extracted for the first raw frame, we could conduct an ultrafast update of features with nearby ROI gradients within several iterations. Across the whole imaging frame sequence, this kind of feature extraction strategy needs only once global image search to cluster features for the first raw frame and template image. Following this initial search, the features of the remaining raw frames can be quickly generated based on the previous information. By contrast, the other methods used global image content (Mitani and Komiyama, 2018); as a result, their computational costs were relatively larger than that of our method.

It is also worth to note that, the features in the raw two-photon images are non-stationary, hence one of the best approaches for feature detection might be performing spatio-temporal feature extraction to tackle temporal inconsistencies. Although computational complexity increases with spatio-temporal information extracted from consecutive frames of imaging data, the processing can preserve both the morphology features and temporal neural dynamics (Luo et al., 2021). As another consequence of non-stationarity, it also poses a challenge for evaluating motion correction, because single metric is hardly able to quantify the performance well. Therefore, a series of complementary metrics in both spatial and temporal dimensions can be combined as the most appropriate solution for measuring the performance quantification, which still needs further investigations.

In this study, we demonstrate that FIFER performed well for motion correction task and required only a conventional computer, such success indicates that it would be desirable to implement FIFER as online processing software and use it for neurobiology experiments requiring online observation and stimulation. However, it warrants to mention that FIFER is designed as a frame-by-frame 2D motion correction method, hence it has the limitation for dealing with the distortions induced by 3D brain movements, which requires 3D motion correction method, e.g., (Griffiths et al., 2020). In addition, although our method demonstrated promising motion correction performance and a fast processing speed in a rigid mode, it would be worthwhile to extend our method to treat affine (Li et al., 2020) or non-rigid distortions (Pnevmatikakis and Giovannucci, 2017) in complex imaging experiments, such as in chronic two-photon imaging to study learning-induced neuronal activity changes.

## Data Availability Statement

The imaging data supporting the conclusions of this article are available from the corresponding author upon reasonable request. The code is provided at https://github.com/Weiyi-Liu-Unique/FIFER. We used a public dataset (the CaImAn dataset file images_N.01.01.zip from https://zenodo.org/record/1659149) to evaluate the performance of FIFER and other methods. For a detailed description of the dataset (see Giovannucci et al., 2019).

## Ethics Statement

This study was approved by the Institutional Animal Care and Use Committee of Third Military Medical University. All experimental procedures were conducted in accordance with animal ethical guidelines of the Third Military Medical University Animal Care and Use Committee.

## Author Contributions

KZ, XC, XnL, and XaL contributed to the design of the study and wrote the manuscript with help from other authors. JP and MW performed the imaging experiments and acquired the data. WL, YX, XnL, and XaL designed the method. WL, JP, YX, HJ, KZ, and XC processed the data sets. All authors contributed to the article and approved the submitted version.

## Conflict of Interest

The authors declare that the research was conducted in the absence of any commercial or financial relationships that could be construed as a potential conflict of interest.

## Publisher’s Note

All claims expressed in this article are solely those of the authors and do not necessarily represent those of their affiliated organizations, or those of the publisher, the editors and the reviewers. Any product that may be evaluated in this article, or claim that may be made by its manufacturer, is not guaranteed or endorsed by the publisher.
